# *APOE* ɛ4, but not polygenic Alzheimer’s disease risk, is related to longitudinal decrease in hippocampal brain activity in non-demented individuals

**DOI:** 10.1038/s41598-023-35316-z

**Published:** 2023-05-24

**Authors:** Sofia Håglin, Elise Koch, Fernanda Schäfer Hackenhaar, Lars Nyberg, Karolina Kauppi

**Affiliations:** 1 Department of Integrative Medical Biology, Umeå University, 901 87 Umeå, Sweden; 2 Umeå Center for Functional Brain Imaging, Umeå University, Umeå, Sweden; 3 Department of Public Health and Clinical Medicine, Umeå University, Umeå, Sweden; 4 Division of Mental Health and Addiction, NORMENT, Centre for Mental Disorders Research, Institute of Clinical Medicine, Oslo University Hospital, University of Oslo, Oslo, Norway; 5 Department of Radiation Sciences, Diagnostic Radiology, University Hospital, Umeå University, Umeå, Sweden; 6 Department of Medical Epidemiology and Biostatistics, Karolinska Institute, Solna, Sweden

**Keywords:** Cognitive ageing, Genetics of the nervous system, Genetics research, Translational research

## Abstract

The hippocampus is affected early in Alzheimer’s disease (AD) and altered hippocampal functioning influences normal cognitive aging. Here, we used task-based functional MRI to assess if the *APOE* ɛ4 allele or a polygenic risk score (PRS) for AD was linked to longitudinal changes in memory-related hippocampal activation in normal aging (baseline age 50–95, n = 292; n = 182 at 4 years follow-up, subsequently non-demented for at least 2 years). Mixed-models were used to predict level and change in hippocampal activation by *APOE* ɛ4 status and PRS based on gene variants previously linked to AD at *p* ≤ 1, *p* < 0.05, or *p* < 5e−8 (excluding *APOE*). *APOE* ɛ4 and PRS_*p*<5e−8_ significantly predicted AD risk in a larger sample from the same study population (n = 1542), while PRS_*p*≤1_ predicted memory decline. *APOE* ɛ4 was linked to decreased hippocampal activation over time, with the most prominent effect in the posterior hippocampi, while PRS was unrelated to hippocampal activation at all p-thresholds. These results suggests a link for *APOE* ɛ4, but not for AD genetics in general, on functional changes of the hippocampi in normal aging.

## Introduction

The biological processes and genetic factors underlying the pathology behind Alzheimer’s disease (AD) and neurocognitive changes in the range of normal aging are partly overlapping^1,2^. Given that the strongest risk factor for AD is increased age, and that early symptoms of AD are difficult to discriminate from aging^1^, it needs to be elucidated how individual variation in normal neurocognitive aging relates to the progression of AD.

AD is considered to have oligogenetic heritability patterns, where the apolipoprotein E (*APOE*) ε4 allele constitutes the single strongest genetic risk factor for the disease^3^. The role of *APOE* in the etiology of AD is not fully understood. Previous studies have linked *APOE* to AD-related mechanisms, including the pathophysiology behind amyloid-β plaques, tau neurofibrils, and neuroinflammation^3^. Recent studies also suggest that *APOE* ε4 may be important for normal cognitive aging independent from AD pathologies^4^.

Several other genetic variants have been associated with AD with weaker effect sizes, linked to genes that are involved in lipid metabolism-, amyloid-*β*-, tau- and immune pathways^5,6^. Polygenic risk scores (PRS) for AD have been used to study the additive effect of multiple gene variants across the whole genome on endophenotypes and to predict disease onset^7^. We have recently shown that both *APOE* ε4 and PRS for AD predicted aging-related cognitive decline across 25 years in individuals that remained healthy at follow-up, where the effect of PRS was seen already six years prior to diagnostic follow-up^8^.

The hippocampus, an important brain area for memory functioning, is structurally and functionally linked to both cognitive aging and development of AD. Atrophy of the hippocampus is observed already at preclinical stages of AD^9^. Both *APOE* ε4 and AD PRS have been linked to hippocampal volume in healthy individuals^10,11^, although a larger study found the effect of AD PRS to be attenuated when removing the *APOE* locus from the PRS^12^. Longitudinal changes in hippocampal volume have been linked to episodic memory decline in *APOE* ε4 carriers but not in non-carriers^13^. fMRI studies of hippocampal activation during memory tasks have shown both hypo- ^14–16^ and hyper-activation^17–22^, i.e. lower or higher amplitude of the fMRI signal, in individuals with mild cognitive impairment (MCI) or AD compared to controls. Altered hippocampal activation has also been reported in cross-sectional studies of healthy *APOE* ε4 allele carriers relative to non-carriers^23–28^. In addition, altered hippocampal activation has been associated with high AD PRS^29,30^.

The aim of the present study was to examine if *APOE* ε4 or AD PRS is associated with level and age-related change in hippocampal activation in healthy individuals. In a previous study using the same data, we observed longitudinal decrease in activation of the anterior hippocampi during memory encoding in aging^31^. Based on this finding, we additionally examined whether a possible association would differ between anterior and posterior hippocampal regions. Our primary hypothesis was that genetic risk factors for AD would magnify the decreases in hippocampal activity previously associated with normal aging.

## Methods

### Participants

Study participants belong to the longitudinal population-based prospective Betula cohort study on memory, health and aging. For an extensive description of the whole Betula project, please see our review by Nyberg et al.^2^. Participants in the Betula study were randomly selected from the population registry in Umeå, Sweden. The aim of the Betula study was to explore memory functioning in adulthood and late life, and to explore early signs and risk factors of dementia. In the Betula project, extensive cognitive data as well as various other variables have been repeatedly assessed 4–5 years apart at six different timepoints (wave 1–6), where brain imaging data is available for wave 5 and wave 6. As previously reported by Nyberg et al.^2^, the memory performance of individuals that underwent fMRI was on average 0.19 standard deviations above that of the total sample of the Betula study, and this difference was more pronounced in older individuals driven by selective attrition before wave 5. Exclusion criteria for the brain scanning sessions were contraindications to MRI or notable artifacts in the fMRI acquisition, history of known neurologic or psychiatric disease, or dementia diagnosis. Individuals who developed dementia until the last diagnostic screening performed in Betula (2015–2017) were excluded from analysis of brain activation (n = 14). A total number of 292 subsequently healthy individuals (141 males and 151 females) aged 50–95 years at the first scanning session (2009–2010) were included, of which 182 returned for a second fMRI scan four years later (2013–2014). To evaluate whether *APOE* ɛ4 carriage and/or a PRS predicts the risk of developing AD, we included a larger sample from the Betula project^2^, of which the brain imaging sample in this study constitutes a subset. PRSs were available for n = 1746 subjects. Subjects with missing data for age and sex (n = 33), unknown dementia diagnosis (n = 116) or with a dementia diagnosis at baseline or before baseline (n = 55) were excluded. Participants with the confounding *APOE* ε2/*APOE* ε4 genotype)^46^ were not included (n = 40). The final sample included 1542 individuals. The study protocol of this project was approved by the local ethics board at Umeå University (Regionala etikprövningsnämnden Umeå, Sweden) and the protocol was followed throughout the study period. All participants provided written informed consent and were compensated monetarily for their participation. The research was performed in accordance with the Declaration of Helsinki.

### Dementia diagnosis procedure

Dementia diagnosis within the whole Betula sample was set in 2015–2017 by a geropsychiatrist based on the DSM-IV criteria^32^, using previous medical history and results from neuroradiological examination. Additional information was obtained from health examination and neuropsychological test assessments as follows: Mini-Mental State Examination (MMSE) score below 23 or a drop by 3 points compared to results from previous test occasion, composite and memory test score ≥ 1.8 standard deviations below age-based norms with a decline in cognitive performance from the previous test occasion, self-reported memory impairment or observed signs of neurocognitive dysfunction at test occasions by nurses and psychologist conducting the testing. Evaluation of medical records was done at baseline and follow-up to increase the diagnostic precision and the reliability of the assessments^33,34^.

### Episodic memory task

A face-name paired-association task including three task conditions; encoding, retrieval, and control, was performed during fMRI acquisition at both baseline and follow-up^35,36^. The face-name stimuli of the encoding condition consisted of an unknown face shown on a black background together with a fictional first name, forming a face-name pair. The names were popular first names, and the faces were digital color photographs with equal numbers of male and female faces. During encoding, participants were asked to remember the face-name pairs and press a button to indicate the item was seen (and to have similar motor responses across the three task conditions). During retrieval, the same faces were presented along with three letters, from which participants were asked to indicate by button press the letter that corresponds to the first letter of the previously encoded name. The left letter corresponded to the index finger, the middle letter to the middle finger, and the right letter to the ring finger. Participants were instructed to guess when not remembering a name. During the active control, participants were asked to indicate with a button press, as quick as possible, when a circle presented at the center of the screen changed to a cross^35,36^. The task was divided into six encoding blocks, six retrieval blocks, and eight blocks of the control task, each 20 s long. For encoding and retrieval, four stimuli were presented per block with a duration of four seconds, separated by the presentation of a cross. The interstimulus intervals was randomized between 1.5 and 4.5 s to allowed for both event-related and blocked analyses. The total fMRI scan time was 10 min for each participant.

### fMRI acquisition

At both baseline and follow-up, the fMRI data were collected on a 3 T General Electric (GE) Discovery MR750 scanner with a 32-channel head coil. The functional images were acquired with a gradient-echo EPI sequence according to the following parameters: TR = 2.0 s, TE = 30 ms (ms), flip angle = 80°, 37 slices (3.9 mm thick), 96 × 96 matrix, FOV = 25 × 25 cm. To allow for saturation of the fMRI signal, ten dummy scans were collected and discarded prior to experimental image acquisition. Cushions inside the head coil were used to minimize subject head movement. E-Prime (Psychology Software Tools) was used for stimulus presentation and recording of responses from a MR-compatible response pad. In addition, structural T1-weighted images were acquired with a 3D fast spoiled gradient echo sequence (180 slices with a 1 mm thickness, TR: 8.2 ms, TE: 3.2 ms, flip angle: 12°, FOV: 25 × 25 cm).

### fMRI analyses

**Figure 1:**
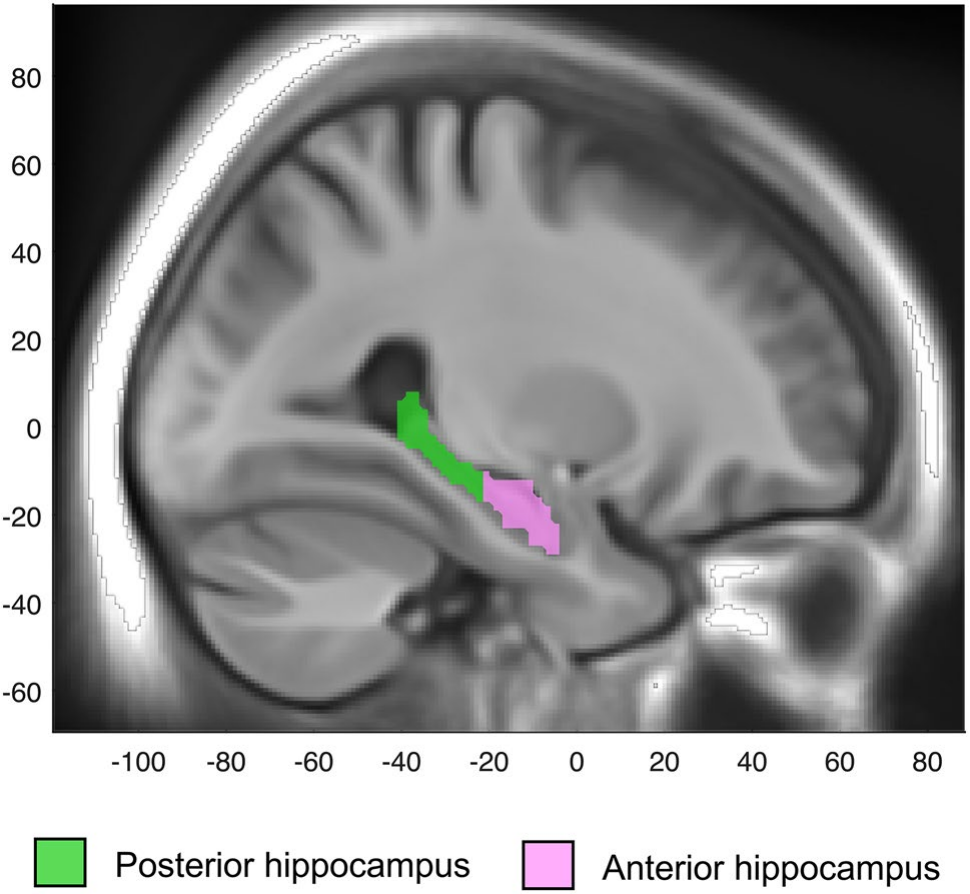
Representation of anterior (purple) and posterior (green) hippocampi according to their location in MNI space relative to y = − 21 mm.

The fMRI data were preprocessed and analyzed using SPM12 (Statistical Parametric Mapping, Wellcome Centre for Human Neuroimaging, http://www.fil.ion.ucl.ac.uk/spm), implemented in MATLAB R2014b (MathWorks). SPM was run through an in-house program (DataZ). Preprocessing of the fMRI data included slice-timing correction, head movement correction by unwarping and realignment to the first image of each volume. The realigned images were then spatially normalized by co-registering participants’ functional images to their structural T1-weighted images. This was done separately for data from baseline and follow-up by segmenting each participants’ structural T1-weighted image into gray matter, white matter and cerebrospinal fluid components. The DARTEL^37^ toolbox was used to create a template image for each participant of the baseline and follow-up data, and also a group-level DARTEL template. The flow-field files that mapped the transformation from native space to DARTEL template and an affine transformation from DARTEL to Montreal Neurologic Institute (MNI) space was used for normalizing the fMRI data to MNI standard space with a 2 mm resolution, and smoothed with an 8 mm full width at half maximum Gaussian kernel. The data were high-pass filtered (128 s), and voxel-wise general linear models were set up for each participant using SPM12. In these models, block onsets and durations for each condition from the scanner task were included as regressor, modeled as a boxcar and convolved with a canonical hemodynamic response function (HRF). To remove movement-related artifacts, six realignment parameters from the motion correction preprocessing steps were included as covariates of no interest. Subject-level contrast images were generated, comparing the experimental conditions of the scanner task (encoding vs. control, and retrieval vs. control), separately for baseline and follow-up data. To identify hippocampal regions more activated during encoding and retrieval relative to the control task, the contrast images were carried on to random-effects group analyses using one sample t-tests of all subjects at baseline. Activation peaks were labeled as either anterior or posterior hippocampus^31^ depending on their location in MNI space relative to y = − 21 mm as suggested by Poppenk et al. for long-axis segmentation of the hippocampus in human neuroimaging^38^, demonstrated in Fig. 1. The coordinates of peak activations were retrieved from fMRI analyses to examine age-related changes in anterior and posterior hippocampus activity at encoding and retrieval, performed in our previous study^31^. Beta values from the hippocampus peak activations, identified from the whole-brain analysis of the baseline sample performed in our previous study^31^, were modeled separately for the left and right hippocampi. Left and right hippocampal volumes were measured using automatic subcortical segmentation (aseg) in FreeSurfer (v. 5.3), and adjusted to head size by dividing the hippocampal volume with the total intracranial volume (ICV).

### Genotyping and construction of polygenic risk scores

The DNA extraction for single-nucleotide polymorphism (SNP) genotyping was done at the Institute of Human Genetics, University of Bonn, Germany. All DNA samples were genotyped using two types of Illumina arrays: Illumina Omni Express and Omni 1S Bead chip kits. Imputation of the raw genotypes was done using the ENIGMA2 protocol of the ENIGMA Consortium (http://enigma.ini.usc.edu/) to the 1000 genomes reference panel^38^ using minimac tools (version 2013.7.17)^39^. Post-imputation quality control (QC) was performed based on genotype call rate < 10%, minor allele frequency (MAF) < 1%, SNP missingness < 5%, and imputation info < 0.8. Before calculating the PRS, SNPs with ambiguous strand alignment were removed, as were SNPs within the *APOE* region (44.4–46.5 Mb on chromosome 19 on the hg19 assembly). Thereafter, PRS for AD were calculated using the summary statistics from a AD GWAS including 21,982 AD cases and 41,944 cognitively normal controls^5^. Linkage disequilibrium (LD) clumping was performed by discarding SNPs within 250 kb of, and in r^2^ ≥ 0.1 with another more significant SNP. The European sample of the 1000 Genomes Project phase 3^40^ was used as LD reference panel for clumping, after removal of SNPs with genotype call rate < 1% and MAF < 1%. PRS were calculated for each individual by summing the alleles of the clumped SNPs weighted by the beta value from the GWAS^5^. PRS were constructed at *p* value thresholds of *p* < 5e−8, *p* < 0.05, and *p* ≤ 1, including 18, 41,305, and 290,660 SNPs, respectively.

### Statistical analyses

To examine if hippocampal activation peaks were associated with scanner task performance or hippocampal volume at baseline, we performed linear regression analyses using the lm function in R. These regression analyses included hippocampal activity as covariate of interest, as well as sex, age, and sample as covariates of no interest. To examine the association of AD PRS and *APOE* ɛ4 with level and change in brain activation in anterior and posterior hippocampus, we performed linear mixed-effect models that were fitted in R using the lmer function available through the lme4 and lmerTest packages, using the extracted beta values as dependent variables. These models included the AD PRS as well as *APOE* ɛ4 carriage status (coded as 0/1 for non-carriers and carriers due to the low frequency of ɛ4 homozygotes) as covariates of interest, and sex, baseline age, adjusted hippocampal volume, sample, education, scanner task performace (number of hits) and the first five genetic principal components (PCs) for genetic ancestry as covariates of no interest. Age at each scanning session was used to estimate the slope, representing individualized aging-related change in activation over five years. Slope of all covariates was included in the models as well as random subject-specific intercepts. To test for association between AD genetics and differences on level and slope of performance on the scanner task (number of hits and response time), a linear mixed-effect model was used, including sex and age at baseline as covariates and *APOE* ɛ4 carriership or PRS as the independent variable and either hits or response time as the dependent variable. As an additional analysis, both behavioral and fMRI analyses of PRS were stratified into *APOE* ɛ4 carriers and non-carriers. All analyses were performed in R version 4.0.3. All continuous variables were z-transformed using the scale function in R, i.e., scaled to zero-mean and standard deviation (SD) of one.

### Genetic prediction of AD risk

To evaluate whether *APOE* ɛ4 and/or PRS predict the risk of AD, we employed a Cause-specific hazard regression accounting for other dementia types and death as competing risks events. These analyses included 1,542 individuals, of which 791 remained healthy, 145 were diagnosed with AD, 121 were diagnosed with other dementia types, and 485 individuals died non-demented. In this type of competing risk regression analysis, the Cause-specific hazard function denotes the instantaneous rate of occurrence of the event (i.e., AD), in participants who are curently event-free. In a sensitivity Cause-specific hazard regression analysis, dementia types other than AD and vascular dementia were excluded due to the low number of cases, such as dementia not-otherwise specified (n = 13), dementia due to Parkinson's disease (n = 8), Lewy body dementia (n = 6), frontotemporal dementia (n = 2), and progressive supranuclear paralysis (n = 1). The sensitivity analysis also excluded individuals with low risk to develop dementia during the studied period (participants younger than 45 years at baseline, n = 184). Thus, sensitivity analysis (n = 1328) included 617 healthy individuals, 145 AD cases, 91 vascular dementia cases and 475 individuals who died non-demented. Cause-specific hazard regression analysis were performed with PRS excluding the *APOE* loci, based on p-thresholds of *p* < 5e−8, *p* < 0.05 and *p* ≤ 1. Time from baseline (in years) was used as the time scale. Regressions were adjusted for the first five PCs, *APOE* ε4 carriage, sex, age, and age-squared, and a sensitivity analysis included years of education. Dementia status, carriers of the *APOE* ε4 allele, and sex were included in the models as binary indicator variables (coded as 0/1).

## Results

### Genetic predictors of AD risk

We tested the effect of *APOE* ε4 carriership and PRS on risk for AD. The PRS was either calculated from all SNPs (PRS_*p*≤1_), SNPs that previously predicted AD at genome-wide significance (PRS_*p*<5e−8_), or SNPs that previously predicted AD at uncorrected significance level (PRS_*p*<0.05_). Both *APOE* ε4 and higher PRS_*p*<5e−8_ were significantly associated with increased risk of AD (Supplementary Table 1, and Supplementary Fig. 1). *APOE* ε4 carriers had a 3.8 times higher risk for AD compared to non-carriers (hazard ratio [HR] = 3.84, CI 2.7–5.4, *p* = 6.8e−15), while an increase in PRS_*p*<5e−8_ by one standard deviation from the mean was associated with a 1.2 times increase in the risk for AD (HR = 1.29, CI 1.080–1.549, *p* = 0.005). PRS_*p*<5e−8_ was similarly associated with risk for AD after controlling for years of education (HR = 1.29, CI 1.08–1.547, *p* = 0.006). In a sensitivity analysis excluding rare dementia types and individuals with low risk for developing AD (younger than 45 years old), the PRS also remained predictive of AD (HR = 1.28, CI 1.073–1.531, *p* = 0.006). PRS_*p*<0.05_ was marginally significantly associated with AD risk, while no effect was seen for PRS_*p*≤1_ (Supplementary Table 1).

### Effects of genetic risk for AD on scanner task performance

Descriptives of the study population undergoing fMRI acquisition are presented in Table 1, sub-grouped according to *APOE* ε4 status. At baseline, there was no relation between *APOE* ε4 or AD PRS at any *p* value threshould with scanner task performance. Longitudinal analyses of scanner task performance revealed that AD PRS_*p*<1_ was significantly associated with more negative slopes in hits across the two fMRI sessions (t = − 3.37, *p* = 0.0008), whereas no effect was seen for *APOE* ε4 (t = − 1.37, *p* = 0.17). The AD PRS had no significant effect at more conservative PRS *p* value thresholds (PRS_*p*<0.05_, t = − 1.65, *p* = 0.0997, PRS_*p*<5e−8_, t = − 0.969, *p* = 0.333). Post-hoc analyses stratified by *APOE* status revealed that the effect of AD PRS_*p*<1_ on slope in task performance was strongest in *APOE* ε4 carriers (n = 78, t = − 2.8, *p* = 0.0056) , with a trend-level effect in non-carriers (n = 213, t = − 1.8, *p* = 0.067). A weak effect of PRS _p<5e-8_ was seen on response time (t = − 2.4, *p* = 0.02, i.e. shorter response time with higher risk), but no other genetic effects were observed for change in response time (all *p* > 0.1).

**Table 1:** Baseline characteristics of study sample for fMRI analyses.

	*APOE* ε4 carriers (n = 77)	*APOE* ε4 non-carriers (n = 215)	t/χ^2^ value	*p* value
Age, Mean (SD)	65.42 (7.96)	67.22 (8.02)	1.69	0.092^a^
Sex, n females (males)	38 (39)	113 (102)	0.12	0.73^b^
BMI	26.55 (3.97)	26.53 (3.66)	− 0.04	1.00^a^
Years of Education, Mean (SD)	13.73 (4.20)	12.18 (4.17)	− 2.79	0.006^a^
MMSE, Mean (SD)	28.16 (1.53)	28.12 (1.45)	− 0.24	0.81^a^
RT, Retrieval, Mean (SD)	2.653 (0.30)	2.673 (0.31)	0.50	0.62^a^
Hits, Retrieval, Mean (SD)	14.29 (3.95)	13.94 (4.24)	− 0.65	0.52^a^
Adj. R Hippocampal volume	2.56e−3	2.52e−3	− 0.89	0.38^a^
Adj. L Hippocampal volume	2.51e−3	2.47e−3	− 0.88	0.38^a^

RT = mean response time (in seconds), SD = standard deviation, BMI = body mass index, Adjusted hippocampal volume = Hippocampal volume/Intracranial volume.

^a^Welch Two Sample t-test,

^b^Chi-Square test. MMSE = Mini mental status evaluation (version 518).

### Effect of genetic risk for AD on age-related change in hippocampal activation

**Figure 2:**
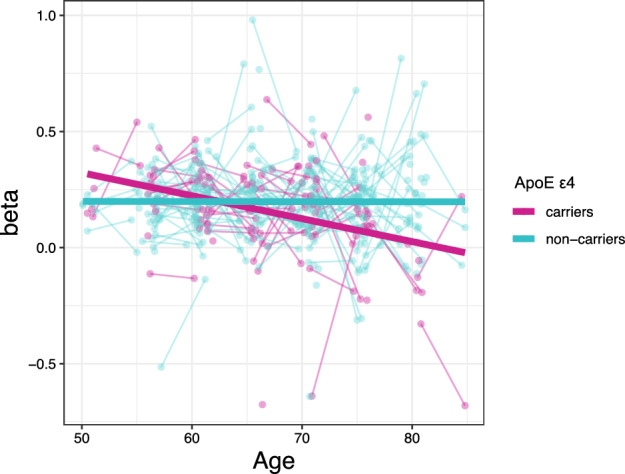
Age-related decrease in hippocampal activity in the left posterior hippocampus during memory encoding in *APOE* ɛ4 carriers (magenta) compared to non-carriers (cyan).

Prior to studies of AD genetics and hippocampal activation, we investigated if hippocampal activation peaks were associated with scanner task performance or hippocampal volume. We found that activation of the right and left anterior as well as the left posterior hippocampi were positively associated with number of hits (Supplementary Table 2), but not with hippocampal volume (Supplementary Table 3). During memory encoding, *APOE* ɛ4 carriers showed a more pronounced aging-related decrease in hippocampal activation relative to non-carriers. The strongest effect was observed in the right and left posterior hippocampi (Table 2, Fig. 2), while weaker effects in the same direction were observed in the anterior hippocampi (Table 2). During memory retrieval, a trend-level effect of *APOE* ɛ4 was seen in the right posterior hippocampus only (Table 2). No effects were observed for any PRS *p* value thresholds on level or slope in hippocampal activation (all *p* > 0.1). An example of the full model is presented in Supplementary Table 4 where the results for the left posteror hippocampus during encoding are shown. Post-hoc stratification based on *APOE* ɛ4 allele carriership or removing scanner task performance (hits and reaction time) from the model did not reveal any significant results of PRS on hippocampal activation (all *p* > 0.1).

**Table 2:** Effect of *APOE* ε4 on level and slope in hippocampal activation across four years.

Contrast, region	Beta	SE	t	*p* value
Encoding				
R posterior				
Intercept	− 4.739e−02	1.843e−02	− 2.571	0.010743*
Slope	− 5.845e−02	1.886e−02	− 3.098	0.002124**
L posterior				
Intercept	− 0.055278	0.020548	− 2.690	0.007647**
Slope	− 0.080679	0.020946	− 3.852	0.000142***
R anterior				
Intercept	− 2.501e−02	2.459e−02	− 1.017	0.31010
Slope	− 4.535e−02	2.493e−02	− 1.819	0.06982
L anterior				
Intercept	− 2.281e−02	2.645e−02	− 0.862	0.38945
Slope	− 6.210e−02	2.682e−02	− 2.315	0.02126*
Retrieval				
R posterior				
Intercept	− 5.904e−02	2.503e−02	− 2.359	0.0192*
Slope	− 5.418e−02	2.521e−02	− 2.149	0.0324*
L posterior				
Intercept	− 4.000e−02	2.707e−02	− 1.478	0.1408
Slope	− 3.852e−02	2.725e−02	− 1.413	0.1585
R anterior				
Intercept	− 7.809e−03	2.942e−02	− 0.265	0.7909
Slope	4.834e−02	3.026e−02	1.598	0.1111
L anterior				
Intercept	− 0.044201	0.029289	− 1.509	0.13265
Slope	0.023385	0.030076	0.778	0.43745

Linear mixed regression models were fitted using the lmer function in R. Sex, baseline age, sample, education, adjusted left or right hippocampus volume, hits, Response time, AD PRS_*p*<1_, and the first 5 genetic principal components for genetic ancestry included in all models. Slope estimated between two time points with a four year interval, using age as time-varying covariate.

SE = standard error, L = left, R = right.

* = *p* < 0.05, ** = *p* < 0.01, *** = *p* < 0.001.

## Discussion

We have studied if the *APOE* ɛ4 allele and/or AD PRS influences aging-related changes in hippocampal functioning across four years, using longitudinal fMRI from a large population-based study^2^. Our hypothesis was that high genetic risk for AD would magnify our previously observed encoding-related decreases in anterior hippocampal activation with aging^31^. We found that *APOE* ɛ4 carriers had decreased hippocampal activation with increasing age, whereas no difference over time was seen in non-carriers (Fig. 2) or as a function of AD PRS. The effect of *APOE* ɛ4 was seen both during encoding and retrieval. The finding did not reflect a magnification of a general effect of age in the anterior hippocampi but was instead expressed most prominently in the posterior hippocampi. O’donogue et al.^41^ reviewed the current literature and described two plausible hypotheses where the role of *APOE* ɛ4 during aging either can be explained as pre-clinical AD pathology (‘prodromal hypothesis’) or direct genetic effects independent of subsequent development of AD (‘phenotypic hypothesis’). In favor of the phenotypic hypothesis, participants in the current study remained non-demented for at least a minimum of two years after the second fMRI assessment, and APOE ɛ4 carriers did not differ from non-carriers in task performance or hippocampal volume. Furthermore, previous fMRI studies have reported a stronger activation of the hippocampi in preclinical AD relative to controls^42,43^, while we instead observed less activation in *APOE* ε4 carriers relative to non-carriers. In addition, the AD PRS excluding the *APOE* locus did not affect hippocampal activity.

A plausible mechanism for an effect of *APOE* ε4 on age-related decreases in hippocampal functioning that is not secondary to prodromal AD, would be the role for *APOE* ε4 on breakdown of the blood–brain barrier (BBB)^44^. The link between APOE and BBB breakdown was recently shown to be specific to the hippocampal region and linked to cognitive decline through neurovascular uncoupling independently of amyloid-β and tau pathologies^4^. Similarly, our observation of a decrease in the BOLD signal in *APOE* ε4 carriers may represent an inability to increase blood flow sufficiently upon increased energy demand, rather than a decreased oxygen demand followed by reduced neuronal activity^45,46^. However, without longer follow-up data or AD-specific biomarkers, we cannot rule out the possibility that our results instead reflect prodromal AD processes, or a mixture. It should also be noted that APOE has a role in several pathways of importance for both AD and age-related cognitive decline, recently described in terms of “the APOE cascade hypothesis”^47^. In this model, the *APOE* ε4 gene variant gives rise to biochemical alterations of the APOE protein, resulting in a cascade of phenotypic effects including neuroinflammation, vascular dysfunction and neuropathology, underlying the clinical outcome^48^.

Although reduced hippocampal activation in healthy elderly has previously been linked to reduced memory performance^31,49,50^, lower activation in *APOE* ε4 was not linked to lower performance on the scanner task in this study. One explanation for this could be that we used a memory task at scanning that was optimized to elicit a strong hippocampal response, but not to reveal behavioral effects. In a previous publication on the same study population, we reported an *APOE* ε4 effect on age-related decreases in performance on more sensitive off-line cognitive tests^8^.

By segmentation of the hippocampi along the anterior–posterior axis, we could further observe that the effect of *APOE* ε4 was most prominent in the posterior hippocampi. However, since *APOE* ε4 also showed a slight effect on the activity in the anterior hippocampi, regional differences should be interpreted cautiously. APOE is expressed mainly in astrocytes and glia cells, but also in neurons of all hippocampal subunits (CA1-4 and DG)^51^. Potential differences of the effect of *APOE* ε4 on the BBB in hippocampal subunits have not yet been explored.

Based on previous studies showing effects of AD PRS on cognitive decline^8^ and level of hippocampal functioning in healthy aging^29,30^, we hypothesized that AD PRS would also predict decline in hippocampal activation. However, we did not observe such an effect for any of our three selected PRS *p* value thresholds. As our PRS sucessfully predicted both AD risk and scanner task performance, the lack of effect on brain activation is unlikely due to low power. Notably, only the PRS_*p*≤1,_ i.e. including all SNPs, predicted decline in task performance across age, in line with our previous work on cognitive task performance^8^. This effect may result from the higher polygenicity of cognitive ability in general than of clinical AD^52,53^. The mechanisms behind variants with weaker association to AD could be mediated through a general effect on cognitive ability that impacts AD risk through educational attainment or other life-style factors. Thus, the genetic link to AD for PRS_*p*≤1_ is seemingly too distant to predict the disease in small independent studies, while a link to cognitive decline is more proximal. In contrast, the PRS_*p*<5e−8_ consisting of 18 SNPs excluding the *APOE* locus, captures genes with roles in AD-related processes, e.g. amyloid-β-, tau-, lipid transportations-, immune system-, and endocytosis pathways^5,6^. Our results suggest that those genes in general can predict AD risk in a small independent sample, but do not influence cognition or hippocampal functioning in healthy aging. However, one or a few of those genetic pathways might still play a role in normal neurocognitive aging individually.

## Limitations

Given that pathological AD processes can start ten years before clinical detection of AD^1^, lack of longer diagnostic follow-up cannot rule out the possibility that some individuals who were assessed as cognitive healthy in fact were in a preclinical dementia stage. However, our two-year clinical follow-up was more extensive than most previous studies on aging in non-demented individuals. The number of individuals that both underwent fMRI and have developed AD in this sample is too small to restrict analyses to this specific sub-group. It should be noted that AD was defined clinically, and that clinically healthy individuals can manifest with AD-related biomarkers in the cerebrospinal fluid that meet the criterias for a *biologically-defined* AD^54^. We have examined a limited set of hippocampal regions. Although we argue that our areas of choice represent relevant parts of the hippocampi affected by aging, it cannot be ruled out that the analyses of additional regions would result in different outcomes.

In conclusion, our findings suggest an effect of *APOE* ɛ4, but not polygenic risk for AD, on longitudinal change in hippocampal functioning in healthy aging.

## Supplementary Information

Supplementary Information.

## Data Availability

The datasets generated and/or analysed during the current study are not publicly available due to lack of ethical permit for sharing sensitive person data, but are available from the corresponding author on reasonable request.
