# Supplementary material for: APOE ɛ4, but not polygenic Alzheimer’s disease risk, is related to longitudinal decrease in hippocampal brain activity in non-demented individuals
Source: Sci Rep. 2023 May 24;13:8433. doi: 10.1038/s41598-023-35316-z (PMC10209089; doi:10.1038/s41598-023-35316-z)
Supplement: Supplementary file 1 — Supplementary Information. [file 41598_2023_35316_MOESM1_ESM.docx]

**Supplementary table 1**. Cause-specific hazard models evaluating APOE ɛ4 and/or PRS in AD risk prediction, accounting for other dementia types and death as competing risks events.

1. Analysis in the full samples (n = 1542 individuals, of which 791 remained healthy, 145 were diagnosed with AD, 121 were diagnosed with other dementia types, and 485 individuals died non-demented).

| Outcome - time to AD | HR | 95% CI | | p-value |  |
| --- | --- | --- | --- | --- | --- |
| AD PRS_p<5e-8_ | 1.293 | 1.080 | 1.549 | 0.005 | ** |
| *APOE* ε4-carriers | 3.844 | 2.739 | 5.394 | < 0.00001 | *** |
| PC1 | 0.982 | 0.828 | 1.164 | 0.831 |  |
| PC2 | 0.901 | 0.759 | 1.070 | 0.236 |  |
| PC3 | 0.856 | 0.704 | 1.041 | 0.120 |  |
| PC4 | 1.000 | 0.834 | 1.200 | 0.996 |  |
| PC5 | 0.884 | 0.631 | 1.239 | 0.475 |  |
| Sex, male | 0.411 | 0.273 | 0.619 | 0.00002 | *** |
| Age at baseline, years | 2.183 | 1.603 | 2.973 | < 0.00001 | *** |
| Age squared | 0.995 | 0.993 | 0.997 | 0.00002 | *** |
|  |  |  |  |  |  |

| Outcome - time to AD | HR | 95% CI | | p-value |  |
| --- | --- | --- | --- | --- | --- |
| AD PRS_p<0.05_ | 1.191 | 0.997 | 1.423 | 0.054 | ** |
| *APOE* ε4-carriers | 3.942 | 2.798 | 5.556 | < 0.00001 | *** |
| PC1 | 0.991 | 0.835 | 1.177 | 0.919 |  |
| PC2 | 0.930 | 0.785 | 1.102 | 0.401 |  |
| PC3 | 0.854 | 0.704 | 1.037 | 0.111 |  |
| PC4 | 1.025 | 0.856 | 1.228 | 0.789 |  |
| PC5 | 0.885 | 0.627 | 1.248 | 0.486 |  |
| Sex, male | 0.391 | 0.260 | 0.589 | < 0.00001 | *** |
| Age at baseline, years | 2.157 | 1.585 | 2.935 | < 0.00001 | *** |
| Age squared | 0.995 | 0.993 | 0.997 | 0.00002 | *** |

| Outcome - time to AD | HR | 95% CI | | p-value |  |
| --- | --- | --- | --- | --- | --- |
| AD PRS_p<1_ | 1.111 | 0.928 | 1,330 | 0.250 |  |
| *APOE* ε4-carriers | 3.807 | 2.710 | 5.348 | < 0.00001 | *** |
| PC1 | 0.988 | 0.831 | 1.174 | 0.887 |  |
| PC2 | 0.942 | 0.793 | 1.118 | 0.492 |  |
| PC3 | 0.855 | 0.704 | 1.038 | 0.114 |  |
| PC4 | 1.021 | 0.852 | 1.225 | 0.819 |  |
| PC5 | 0.892 | 0.634 | 1.255 | 0.510 |  |
| Sex, male | 0.391 | 0.260 | 0.589 | < 0.00001 | *** |
| Age at baseline, years | 2.172 | 1.594 | 2.960 | < 0.00001 | *** |
| Age squared | 0.995 | 0.993 | 0.998 | 0.00002 | *** |

1. Sensitivity analysis adjusting for years of education in the full sample (n = 1542 individuals, of which 791 remained healthy, 145 were diagnosed with AD, 121 were diagnosed with other dementia types, and 485 individuals died non-demented).

| Outcome - time to AD | HR | 95% CI | | p-value |  |
| --- | --- | --- | --- | --- | --- |
| AD PRS_p<5e-8_ | 1.290 | 1.076 | 1.547 | 0.006 | ** |
| *APOE* ε4-carriers | 3.895 | 2.772 | 5.475 | < 0.00001 | *** |
| PC1 | 0.959 | 0.804 | 1.142 | 0.636 |  |
| PC2 | 0.894 | 0.753 | 1.063 | 0.205 |  |
| PC3 | 0.878 | 0.719 | 1.073 | 0.204 |  |
| PC4 | 1.001 | 0.834 | 1.201 | 0.992 |  |
| PC5 | 0.902 | 0.643 | 1.267 | 0.553 |  |
| Sex, male | 0.411 | 0.272 | 0.620 | 0.00002 | *** |
| Years of education | 1.021 | 0.964 | 1.081 | 0.483 |  |
| Age at baseline, years | 2.201 | 1.610 | 3.009 | < 0.00001 | *** |
| Age squared | 0.995 | 0.993 | 0.997 | 0.00002 | *** |

| Outcome - time to AD | | HR | 95% CI | | p-value |  |
| --- | --- | --- | --- | --- | --- | --- |
| AD PRS_p<0.05_ | 1.181 | | 0.986 | 1.414 | 0.071 | . |
| *APOE* ε4-carriers | 3.979 | | 2.819 | 5.616 | < 0.00001 | *** |
| PC1 | 0.969 | | 0.812 | 1.156 | 0.726 |  |
| PC2 | 0.922 | | 0.778 | 1.094 | 0.352 |  |
| PC3 | 0.873 | | 0.716 | 1.065 | 0.181 |  |
| PC4 | 1.025 | | 0.855 | 1.229 | 0.791 |  |
| PC5 | 0.902 | | 0.639 | 1.274 | 0.559 |  |
| Sex, male | 0.393 | | 0.261 | 0.592 | < 0.00001 | *** |
| Years of education | 1.016 | | 0.959 | 1.076 | 0.590 |  |
| Age at baseline, years | 2.171 | | 1.589 | 2.966 | < 0.00001 | *** |
| Age squared | 0.995 | | 0.993 | 0.998 | 0.00003 | *** |

| Outcome - time to AD | HR | 95% CI | | p-value |  |
| --- | --- | --- | --- | --- | --- |
| AD PRS_p<1_ | 1.110 | 0.926 | 1.331 | 0.259 |  |
| *APOE* ε4-carriers | 3.856 | 2.740 | 5.426 | < 0.00001 | *** |
| PC1 | 0.967 | 0.809 | 1.154 | 0.708 |  |
| PC2 | 0.934 | 0.786 | 1.110 | 0.439 |  |
| PC3 | 0.874 | 0.717 | 1.066 | 0.184 |  |
| PC4 | 1.022 | 0.852 | 1.226 | 0.816 |  |
| PC5 | 0.908 | 0.644 | 1.280 | 0.583 |  |
| Sex, male | 0.393 | 0.261 | 0.592 | < 0.00001 | *** |
| Years of education | 1.016 | 0.959 | 1.076 | 0.598 |  |
| Age at baseline, years | 2.182 | 1.595 | 2.984 | < 0.00001 | *** |
| Age squared | 0.995 | 0.993 | 0.998 | 0.00003 | *** |

1. Sensitivity analysis with vascular dementia and death as competing risk events (i.e., no rare dementia types), and excluding subjects with low risk of developing dementia during the study period (i.e., no younger than 45 years at baseline). n = 1328 individuals, of which 617 remained healthy, 145 were diagnosed with AD, 91 were diagnosed with vascular dementia and 475 individuals died non-demented.

| Outcome - time to AD | HR | 95% CI | | p-value |  |
| --- | --- | --- | --- | --- | --- |
| AD PRS_p<5e-8_ | 1.282 | 1.073 | 1.531 | 0.006 | ** |
| *APOE* ε4-carriers | 3.748 | 2.671 | 5.259 | < 0.00001 | *** |
| PC1 | 0.984 | 0.830 | 1.167 | 0.856 |  |
| PC2 | 0.896 | 0.754 | 1.065 | 0.213 |  |
| PC3 | 0.862 | 0.709 | 1.048 | 0.137 |  |
| PC4 | 1.013 | 0.844 | 1.216 | 0.891 |  |
| PC5 | 0.922 | 0.692 | 1.227 | 0.578 |  |
| Sex, male | 0.412 | 0.274 | 0.620 | 0.00002 | *** |
| Age at baseline, years | 2.161 | 1.580 | 2.956 | < 0.00001 | *** |
| Age squared | 0.995 | 0.993 | 0.998 | 0.00003 | *** |

| Outcome - time to AD | HR | 95% CI | | p-value |  |
| --- | --- | --- | --- | --- | --- |
| AD PRS_p<0.05_ | 1.189 | 0.996 | 1.420 | 0.056 | . |
| *APOE* ε4-carriers | 3.859 | 2.737 | 5.441 | < 0.00001 | *** |
| PC1 | 0.993 | 0.836 | 1.178 | 0.933 |  |
| PC2 | 0.922 | 0.778 | 1.094 | 0.354 |  |
| PC3 | 0.858 | 0.707 | 1.042 | 0.122 |  |
| PC4 | 1.039 | 0.866 | 1.245 | 0.683 |  |
| PC5 | 0.924 | 0.691 | 1.237 | 0.597 |  |
| Sex, male | 0.396 | 0.263 | 0.595 | < 0.00001 | *** |
| Age at baseline, years | 2.134 | 1.562 | 2.915 | < 0.00001 | *** |
| Age squared | 0.995 | 0.993 | 0.998 | 0.00005 | *** |

| Outcome - time to AD | HR | 95% CI | | p-value |  |
| --- | --- | --- | --- | --- | --- |
| AD PRS_p<1_ | 1.105 | 0.927 | 1.318 | 0.266 |  |
| *APOE* ε4-carriers | 3.727 | 2.652 | 5.237 | < 0.00001 | *** |
| PC1 | 0.990 | 0.833 | 1.176 | 0.908 |  |
| PC2 | 0.934 | 0.786 | 1.111 | 0.442 |  |
| PC3 | 0.860 | 0.709 | 1.044 | 0.128 |  |
| PC4 | 1.035 | 0.862 | 1.241 | 0.715 |  |
| PC5 | 0.930 | 0.696 | 1.242 | 0.621 |  |
| Sex, male | 0.395 | 0.263 | 0.594 | < 0.00001 | *** |
| Age at baseline, years | 2.150 | 1.571 | 2.942 | < 0.00001 | *** |
| Age squared | 0.995 | 0.993 | 0.998 | 0.00004 | *** |

AD, Alzheimer's disease. APOE, apolipoprotein E. CI, confidence interval. PC, principal component. HR, hazard ratio. PRS, polygenic risk score. . = p < 0.1, * = p < 0.05, ** = p < 0.01, *** = p < 0.001.

**Supplementary Figure 1:**


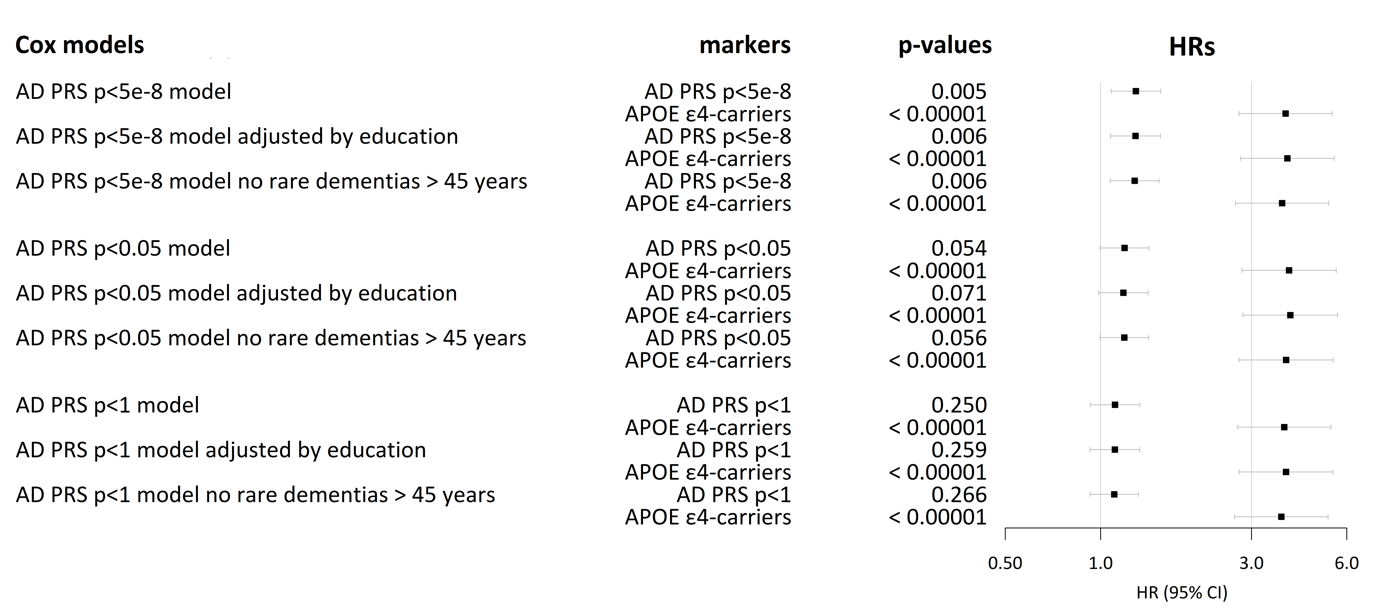


**Figure S1**. Forest plot summarizing results from Cause-specific hazard models evaluating APOE ɛ4 and/or PRS in AD risk prediction from Table S1. HR= hazard ratios.

**Supplementary Table 2.** Relationship between hippocampal peaks and scanner task performance at baseline

|  |  | **beta** | **SE** | **t-value** | **p-value** |
| --- | --- | --- | --- | --- | --- |
| **RT, Retrieval** |  |  |  |  |  |
| Encoding | R anterior | -135.65 | 78.58 | -1.726 | 0.085 |
|  | L anterior | -117.22 | 73.04 | -1.605 | 0.120 |
|  | R posterior | -42.05 | 103.92 | -0.405 | 0.686 |
|  | L posterior | -164.10 | 96.04 | -1.709 | 0.089 |
| Retrieval | R anterior | 1.171 | 66.03 | 0.018 | 0.986 |
|  | L anterior | -81.98 | 61.62 | -1.330 | 0.184 |
|  | R posterior | -43.08 | 68.39 | -0.630 | 0.529 |
|  | L posterior | -36.83 | 67.24 | -0.548 | 0.584 |
| **Hits, Retrieval** |  |  |  |  |  |
| Encoding | R anterior | 2.202 | 0.963 | 2.286 | 0.023* |
|  | L anterior | 2.238 | 0.893 | 2.506 | 0.013* |
|  | R posterior | 1.197 | 1.277 | 0.937 | 0.350 |
|  | L posterior | 4.040 | 1.164 | 3.472 | 0.0006* |
| Retrieval | R anterior | 1.861 | 0.805 | 2.311 | 0.022* |
|  | L anterior | 2.051 | 0.751 | 2.732 | 0.007* |
|  | R posterior | 1.446 | 0.838 | 1.725 | 0.086 |
|  | L posterior | 1.852 | 0.821 | 2.265 | 0.025* |

RT = mean response time (in seconds). SE = standard error. R = right. L = left. Linear regression analyses included sex, baseline age, and sample as covariates of no interest. ﻿* = p < 0.05

**Supplementary Table 3**: Relationship between hippocampal peaks and hippocampal volume at baseline

|  |  | **beta** | **SE** | **t-value** | **p-value** |
| --- | --- | --- | --- | --- | --- |
| **Adj. R Hippocampal volume** |  |  |  |  |  |
| Encoding | R anterior | -9.16e-5 | 8.06e-5 | -1.136 | 0.257 |
|  | L anterior | -8.25e-5 | 7.47e-5 | 1.104 | 0.270 |
|  | R posterior | 2.83e-5 | 1.07e-4 | 0.265 | 0.791 |
|  | L posterior | 8.72e-5 | 9.91e-5 | 0.880 | 0.380 |
| Retrieval | R anterior | -6.88e-5 | 6.94e-5 | -0.991 | 0.323 |
|  | L anterior | 1.53e-5 | 6.40e-5 | 0.239 | 0.811 |
|  | R posterior | 9.59e-5 | 7.94e-5 | 1.208 | 0.228 |
|  | L posterior | 1.14e-4 | 7.49e-5 | 1.520 | 0.130 |
| **Adj. L Hippocampal volume** |  |  |  |  |  |
| Encoding | R anterior | -1.50e-4 | 8.07e-5 | -1.858 | 0.064 |
|  | L anterior | -1.20e-4 | 7.49e-5 | -1.602 | 0.110 |
|  | R posterior | -6.01e-6 | 1.07e-4 | -0.056 | 0.955 |
|  | L posterior | 3.91e-5 | 9.97e-5 | 0.392 | 0.695 |
| Retrieval | R anterior | -9.18e-5 | 6.97e-5 | -1.317 | 0.189 |
|  | L anterior | 5.32e-5 | 6.42e-5 | 0.828 | 0.408 |
|  | R posterior | 9.77e-5 | 7.98e-5 | 1.224 | 0.222 |
|  | L posterior | 1.44e-4 | 7.51e-5 | 1.912 | 0.057 |

SE = standard error. R = right. L = left. Adjusted hippocampal volume = Hippocampal volume/Intracranial volume. Linear regression analyses included sex, baseline age, and sample as covariates of no interest. ﻿

| **Supplementary table 4.** Full model of AD genetics in relation to brain activation in the right posterior hippocampus during the contrast Encoding -baseline. | | | | | |  |
| --- | --- | --- | --- | --- | --- | --- |
|  |  |  |  |  |  |  |
|  | **Estimate** | **SE** | **df** | **t** | **p** |  |
|  |  |  |  |  |  |  |
| (Intercept) | 1.736e-01 | 4.644e-02 | 2.506e+02 | 3.739 | 0.000229 |  |
| scale(Inclusion_age) | 2.198e-02 | 3.211e-02 | 2.968e+02 | 0.685 | 0.494084 |  |
| Education | -8.376e-06 | 2.366e-03 | 2.457e+02 | -0.004 | 0.997178 |  |
| scale(Age) | -2.329e-02 | 5.294e-02 | 4.318e+02 | -0.440 | 0.660270 |  |
| sample | -9.209e-03 | 5.644e-03 | 2.724e+02 | -1.632 | 0.103887 |  |
| sex | 2.075e-02 | 1.795e-02 | 2.651e+02 | 1.155 | 0.248936 |  |
| scale(R_Hippo_ICVcontroll) | 1.673e-02 | 9.630e-03 | 2.689e+02 | 1.737 | 0.083490 |  |
| scale(FN_hits) | 3.236e-03 | 1.019e-02 | 4.105e+02 | 0.317 | 0.751069 |  |
| scale(FN_Ret_RT) | -5.679e-03 | 9.337e-03 | 3.941e+02 | -0.608 | 0.543346 |  |
| scale(PC1) | -8.771e-03 | 8.767e-03 | 2.460e+02 | -1.001 | 0.318043 |  |
| scale(PC2) | 8.146e-03 | 8.279e-03 | 2.428e+02 | 0.984 | 0.326081 |  |
| scale(PC3) | 6.741e-03 | 8.267e-03 | 2.584e+02 | 0.815 | 0.415640 |  |
| scale(PC4) | 7.709e-03 | 8.186e-03 | 2.411e+02 | 0.942 | 0.347305 |  |
| scale(PC5) | 2.508e-03 | 8.308e-03 | 2.546e+02 | 0.302 | 0.763040 |  |
| scale(PRS_p1) | -3.353e-03 | 8.716e-03 | 2.491e+02 | -0.385 | 0.700773 |  |
| APOE e4 | -4.739e-02 | 1.843e-02 | 2.395e+02 | -2.571 | 0.010743 |  |
| Education:scale(Age) | 8.389e-04 | 2.529e-03 | 3.234e+02 | 0.332 | 0.740286 |  |
| scale(Age):sample | 1.356e-03 | 4.725e-03 | 3.130e+02 | 0.287 | 0.774343 |  |
| scale(Age):sex | -4.209e-03 | 1.736e-02 | 3.205e+02 | -0.242 | 0.808591 |  |
| scale(Age):scale(RHC_vol) | -4.718e-03 | 8.503e-03 | 3.210e+02 | -0.555 | 0.579396 |  |
| scale(Age):scale(FN_hits) | -1.552e-02 | 8.985e-03 | 4.268e+02 | -1.728 | 0.084772 |  |
| scale(Age):scale(FN_RT) | 9.831e-03 | 8.912e-03 | 4.279e+02 | 1.103 | 0.270602 |  |
| scale(Age):scale(PC1) | -9.090e-03 | 8.668e-03 | 2.998e+02 | -1.049 | 0.295167 |  |
| scale(Age):scale(PC2) | 2.678e-03 | 8.723e-03 | 3.068e+02 | 0.307 | 0.759023 |  |
| scale(Age):scale(PC3) | -1.286e-03 | 8.459e-03 | 3.256e+02 | -0.152 | 0.879229 |  |
| scale(Age):scale(PC4) | 3.198e-03 | 8.542e-03 | 3.066e+02 | 0.374 | 0.708387 |  |
| scale(Age):scale(PC5) | -8.651e-03 | 8.367e-03 | 3.071e+02 | -1.034 | 0.302011 |  |
| scale(Age):scale(PRS_p1) | -3.485e-03 | 8.239e-03 | 3.373e+02 | -0.423 | 0.672604 |  |
| scale(Age):APOE e4 | -5.845e-02 | 1.886e-02 | 3.125e+02 | -3.098 | 0.002124 |  |
